# Nanoparticles engineered to bind cellular motors for efficient delivery

**DOI:** 10.1186/s12951-018-0354-1

**Published:** 2018-03-30

**Authors:** Inmaculada Dalmau-Mena, Pablo del Pino, Beatriz Pelaz, Miguel Ángel Cuesta-Geijo, Inmaculada Galindo, María Moros, Jesús M. de la Fuente, Covadonga Alonso

**Affiliations:** 1 Dpt. Biotecnología, Instituto Nacional de Investigación y Tecnología Agraria y Alimentaria (INIA), Carretera de la Coruña km 7.5, 28040 Madrid, Spain; 2 Instituto de Nanociencia de Aragón, Universidad de Zaragoza, Mariano Esquillor, s/n, 50018 Zaragoza, Spain; 3 Centro Singular de Investigación en Química Biolóxica e Materiais Moleculares (CiQUS), Departamento de Física de Partículas, Universidad de Santiago de Compostela, 15782 Santiago de Compostela, Spain; 4 Aragon Materials Science Institute (ICMA), CSIC-University of Zaragoza and CIBER-BBN, C/Pedro Cerbuna 12, 50009 Zaragoza, Spain

**Keywords:** Nanoparticles, Biomimetic synthetic peptides, Drug delivery, Microtubule motors, Dynein

## Abstract

**Background:**

Dynein is a cytoskeletal molecular motor protein that transports cellular cargoes along microtubules. Biomimetic synthetic peptides designed to bind dynein have been shown to acquire dynamic properties such as cell accumulation and active intra- and inter-cellular motion through cell-to-cell contacts and projections to distant cells. On the basis of these properties dynein-binding peptides could be used to functionalize nanoparticles for drug delivery applications.

**Results:**

Here, we show that gold nanoparticles modified with dynein-binding delivery sequences become mobile, powered by molecular motor proteins. Modified nanoparticles showed dynamic properties, such as travelling the cytosol, crossing intracellular barriers and shuttling the nuclear membrane. Furthermore, nanoparticles were transported from one cell to another through cell-to-cell contacts and quickly spread to distant cells through cell projections.

**Conclusions:**

The capacity of these motor-bound nanoparticles to spread to many cells and increasing cellular retention, thus avoiding losses and allowing lower dosage, could make them candidate carriers for drug delivery.

**Electronic supplementary material:**

The online version of this article (10.1186/s12951-018-0354-1) contains supplementary material, which is available to authorized users.

## Background

Motor proteins are biological molecules that transform chemical energy into mechanical force at the nanoscale [1]. These molecular motors can be integrated into hybrid biological and synthetic systems to functionalize nanoparticles (NPs) for biotechnological applications [2].

Engineered nanocomposites have been built in order to overcome intracellular barriers and to mimic how intracellular pathogens such as viruses transfer their genetic material into the cells [3]. Several design strategies have been tested in order to increase NPs translocation through cellular membranes, thereby enhancing transfection efficiency. Ligands conjugated to the surface of engineered NPs such as sugars [4] can influence the mode of cellular internalization [5]. Nevertheless, NPs of the same composition may use alternative mechanisms for cell entry in different cell types [6].

Once inside the cell and given the crowded gel-like composition of the cytoplasm, the movement of organelles and macromolecules is restricted [7]. Furthermore, eukaryotic cells are compartmentalized by endomembrane systems and cytoskeleton filaments of actin, and microtubules are used as tracks for transport. This transport relies on energy-dependent molecular motors that are necessary for communication between compartments. To move in a crowded cellular environment poses problems to NP displacement and thus, vector design strategies have included different mediators of intracellular trafficking in order to facilitate this transport, some of them considering the cytoskeleton [8–10]. In fact, viruses, strictly depending on their capacity to invade and control the intracellular environment, have developed specific strategies to bind microtubule motors to facilitate their transport along these filaments to reach the perinuclear area and initiate replication [11–15]. Microtubule motor binding is crucial for the replication and dissemination of several viruses. In-depth knowledge of some of these strategies of virus-cell interaction can be exploited to design virus-free delivery systems [16–18].

We used a biomimetic approach based on selected peptide sequences from viral proteins known to bind dynein. Dynein is a microtubule motor acting as a hub protein within the microtubule-motor complex, which exerts several functions related to movement in mammalian cells. This protein is responsible for cargo transport within cells, up to organelle-size cargoes [19]. It is also responsible of nuclear positioning within the cell and has several functions in mitosis [20–22]. Onset of mitosis is marked by centrosome migration for the mitotic spindle formation that requires dynein. Also, dynein action on microtubules leads to nuclear envelope breakdown at early mitosis and to its reorganization after the end of cell division [23–25]. The loss of nuclear compartmentalization induced by the breakdown of the nuclear envelope is characterized by increased permeability together with the initiation of the nuclear pore disassembly [26]. Thus, dynein has the potential capability to drive nuclear envelope disassembly and permeate this layer, that is, a particularly important property since the nuclear membrane is the “last barrier” for gene delivery in non-dividing cells.

Dynein exists within a complex of several subunits and adapters [27] and contains two heavy chains and several intermediate, light intermediate and light chains [28, 29]. One of the light chains is DYNLL1, a highly evolutionarily conserved protein that is hijacked by a number of viral proteins for transport [30]. The high affinity interaction of viral proteins with DYNLL1 [31] and its ability to form long half-live molecular complexes make this molecule especially appropriate to build supramolecular structures [32]. Then, we used peptide sequences from viral proteins interacting with DYNLL1 to modify NPs based in the hypothesis that this modification could confer dynamic properties to the NPs, enhancing their motility and dispersion in the intracellular environment.

In the present work, gold NPs (Au NPs) have been modified with short peptide sequences taken from viral proteins that bind dynein. We found a highly active intracellular trafficking and cellular uptake of those modified carriers in several cell lines. Dynein-binding peptides (DBPs) modified NPs were motile throughout the cytosol and travelled from cell-to-cell favoring the propagation of this uptake. By this active cellular transport, NPs acquired the property to shuttle the nuclear membrane in non-dividing cells.

## Results

### Dynein-binding peptides modified nanoparticles design

**Fig. 1:**
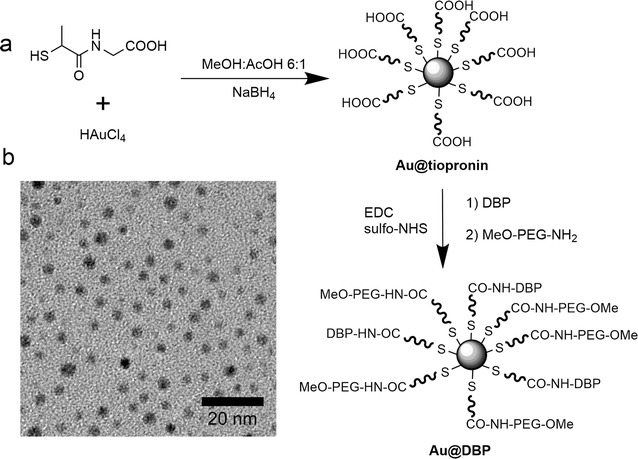
**a** Scheme of NPs synthesis (Au@tiopronin) and functionalization with dynein-binding peptides (DBP) and PEG. **b** TEM image of Au@tiopronin. Scale bar: 20 nm

Here, we report the production of water-soluble and stable Au NPs modified with DBPs as an efficient approach for cargo delivery. Au NPs of different sizes have been widely used because of lack of apparent toxicity, and easy elimination by renal clearance in case NPs are small enough (sizes bellow 10 nm) [33, 34]. Coating NPs with organic molecules and/or macromolecules has been typically employed in order to improve NP stability and prevent aggregation [33], as for instance Au NPs coated with tiopronin (Au@tiopronin) [35]. Tiopronin is a thiolated derivative of the amino acid glycine. Au@tiopronin were prepared using the procedure originally reported by Murray et al. [34]. These NPs consist of small (typically, core diameter < 3 nm) Au NPs stabilized with the non-natural aminoacid tiopronin (*N*-2-mercaptopropionylglycine), which has a free terminal carboxyl group, allowing to functionalize Au@tiopronin with aminated molecules [35], such as the DBPs sequences, by carbodiimide chemistry, *cf*. Figure 1. The reaction was executed in a methanolic/acetic acid mixture, dissolving HAuCl_4_ and tiopronin to give a stable solution. The addition of NaBH_4_ as reducing agent provided a dark solution by reduction of the gold salt and formation of the NPs. Acidic conditions are very important to guarantee the protonation of tiopronin carboxylic groups, and provide an efficient and dense self-assembled monolayer of tiopronin onto the NPs. The obtained NPs were colloidally stable in aqueous solution. The excess of tiopronin and salts was eliminated by dialysis. The purified NPs were characterized by TEM and UV/Vis, *cf*. Additional file 1: Figure S1. TEM images showed a mean diameter of 2.8 nm for the Au core (Fig. 1a). The UV/Vis absorption spectra showed an almost non-detectable surface plasmon band consistent with the small NP size of the NPs, *cf.*, Additional file 1: Figure S1.

Using the reactivity of the tiopronin carboxylic group, further functionalization with aminated molecules was carried out using a procedure previously described [35] (Fig. 1): 1) Aminated poly(ethylene glycol) (PEG) derivative (CH_3_O-PEG-NH_2_); 2) different peptide sequences (Table 1) and 3) fluorescent dye TAMRA-CAD (*i.e.*, tetramethylrhodamine 5-carboxamide cadaverine), which was used to optimize carbodiimide coupling conditions. PEG molecules were selected to avoid unspecific interactions. A dense layer of PEG confers anti-fouling properties to nanomaterials, thereby increasing their colloidal stability in aqueous solution and reducing unspecific interactions with other molecules such as plasma proteins [36–38].

**Table 1:** Alias, sequence and molecular weight (MW) of the peptides used to surface modify the Au@tiopronin NPs

Alias	Amino acid sequence	MW (Da)
DynPro	GGGGK(TAMRA)-HPAEPGSTVTTQNTASQTMS-RRRRRRRR	4063.5
ShortPro	GGGGGGGGK(TAMRA)-YTTQNTASQTMS-RRRRRRRR	3578.9
TrasnRb	GGGGGGGGK(TAMRA)-FPNPSGRSSEDKSTQTAG-RRRRRRRR	4112.5
IntCt	GGGGK(TAMRA)-SLVSSDESVLHGSHESGEHV-RRRRRRRR	4110.5

The selected dynein-binding peptide (DBP) sequences were synthetic peptides spanning the dynein-binding domain of different proteins, *cf.* Table 1. We selected three peptides: DynPro (dynein-propelled) derived from the product of the E183L gene of the African swine fever virus (p54) [11, 31]; ShortPro, a shorter sequence of the same protein based on the critical amino acids for dynein-binding; and TransRb derived from the Rabies virus P protein [39]. As a negative control, a non-dynein-binding amino acid sequence (IntCt) was selected. To guarantee the proper presentation of the peptides and minimize steric hindrance due to a dense packaging of molecules on the NP surface, a tetra- or octa-glycine tail was added to DynPro and IntCt, or ShortPro and TransRb, respectively. After the glycine-tail, a lysine residue was added. Primary amine moieties of the lysine residues were used for fluorescent labeling with 5-carboxytetramethylrhodamine (TAMRA). Finally, an octa-arginine tail was added to these peptides’ end, including to the control peptide IntCt, to increase the positive charge of the peptides, thereby enhancing cellular uptake, *cf.* Table 1.

NPs´carboxyl groups and primary amines of the selected ligands (PEG derivative, TAMRA-CAD and DBPs) were crosslinked with the water-soluble carbodiimide N-[3-(dimethylamino)propyl]-N’-ethylcarbodiimide hydro-chloride (EDC). N-hydroxysulfosuccinimide (sulfo-NHS) was also included in the reaction mixture to improve the efficiency of the carbodiimide-mediated amide-forming reaction by producing hydrolysis-resistant active ester reaction intermediates (Fig. 1). The resultant library of NPs was analyzed by ζ-potential, UV/Vis and fluorescence spectroscopy, probing that the molecules were incorporated to the NPs and the absence of aggregation after peptide coupling, *cf*. Additional file 1: Figure S3.

### Modified nanoparticles internalization

**Fig. 2:**
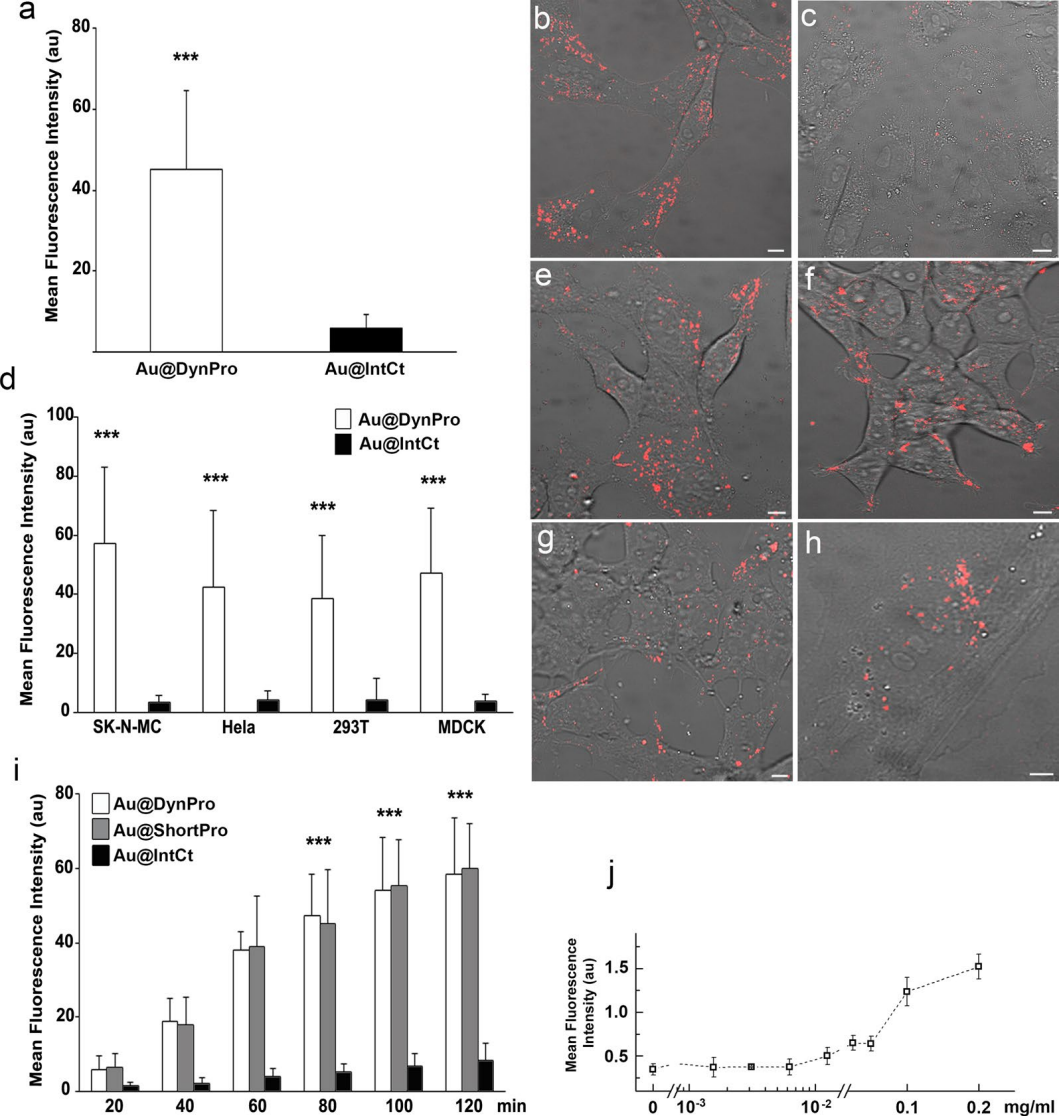
Cellular distribution of NPs. **a**, **d** Mean fluorescence intensity (MFI) of intracellular Au@tiopronin modified with DynPro (Au@DynPro) or IntCt (Au@IntCt) after 1 h incubation with Vero cells (**a**) and other cell lines (**d**). Differences in MFI between Au@DBPs and Au@IntCt (control) were statistically significant *p* value of 0.001 (α = 0.05). In contrast, differences among Au@DBPs were not significant with a *p* value of 0.3 (α = 0.05; not shown). **b** Representative confocal images of Vero cells incubated with Au@DynPro or **c** Au@IntCt. **e**–**h** Representative confocal images of SK-N-MC (**e**), HeLa (**f**), 293T (**g**) and MDCK cells incubated with Au@DynPro. Scale bar: 10 µm. **i** Time-dependent accumulation of Au@DynPro at several time points between 20 and 120 min, as indicated. **j** Fluorescence intensity percentages at increasing doses of Au@DynPro quantified by flow cytometry

**Fig. 3:**
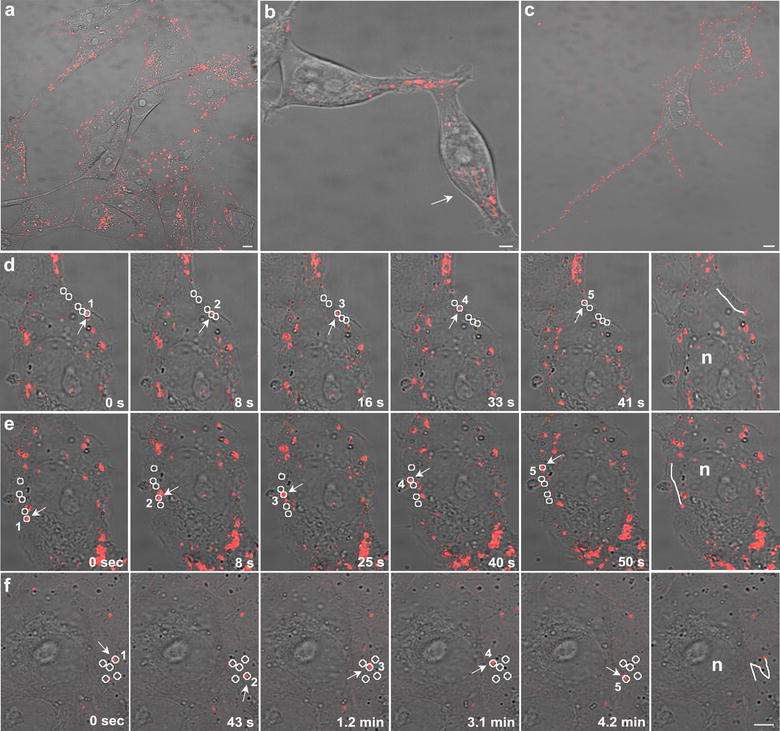
Representative confocal images at low magnification of **a** Vero cells with Au@DynPro **b** HEK293T cells with Au@ShortPro and **c** MDCK cells with Au@TransRb. Representative images showed similar NP dispersion throughout the culture and cell-to-cell transfer of Au@DynPro through short (**b**) or long (**c**) projections. **d**, **e** Representative time-lapse images of the linear progression of Au@DBP inside the cell and the resulting trajectories (circles). **f** Comparison with the non-linear movement obtained with control NPs Au@IntCt. Scale bar: 5 µm

NPs covalently functionalized with TAMRA-labeled peptides were visualized by live-cell imaging, which allowed tracking of their movement at the intracellular level. The cellular uptake of Au@tiopronin NPs modified with DBPs (Au@DBPs; Fig. 2b, e–h) was significantly more efficient compared to those functionalized with the control peptide IntCt (*i.e.*, Au@IntCt) in Vero cells (p < 0.001; Fig. 2c). Similar results were obtained with other cell lines, such as the neuroblastoma cell line SK-N-MC, the epithelial cell line MDCK, HeLa (derived from a human epithelial carcinoma) and HEK293T cell line (human kidney embryonic transformed cell line; Fig. 2d–h). Time-dependent intracellular accumulation was observed from 20 to 120 min with Au@DBPs and Au@IntCt (Fig. 2i). Concentration of Au@DBPs increased until 180 min, remaining stable thereafter and visible up to 12 h. Quantitative fluorescence percentages were also analyzed by flow cytometry with increasing doses of NPs (Fig. 2j). Overall, the cellular distribution among the functionalized Au@DBPs was similar (Fig. 3a–c). Au@DBPs were rapidly internalized and distributed widely within the cell and throughout the culture. While NPs stability in cell culture media was clearly observed, PEG modification did not result in significant modification of Au@DBPs uptake in cells incubated with 0.2 mg/ml NPs (ca. 1.5 μM NPs) for several times 1, 2 or 3 h (Additional file 1: Figure S4).

### Nanoparticles dynamic properties

Time-lapse videomicroscopy unraveled Au@DBP bidirectional movement across the cytosol and a continuous flow to cell projections reaching neighboring or distant cells (Fig. 3a-c and Additional file 2: Movie 1). Also, we observed bidirectional movement towards the cell periphery, to cell projections and intercellular cell-to-cell transport. No differences were found among Au@DBPs in terms of distribution and dynamic properties (Fig. 3a–c, Additional file 2: Movies 1, Additional file 3: Movies 2). Au@DBPs exhibited high-speed mobility (median speed 0.25 ± 0.2 µm/s). This movement followed linear trajectories allowing progression of Au@DBPs (Fig. 3d, e). In contrast, the movement of NPs functionalized with control peptide (Au@IntCt) was non-progressive, non-linear (Fig. 3f).

### Microtubule-dependent transport of nanoparticles

**Fig. 4:**
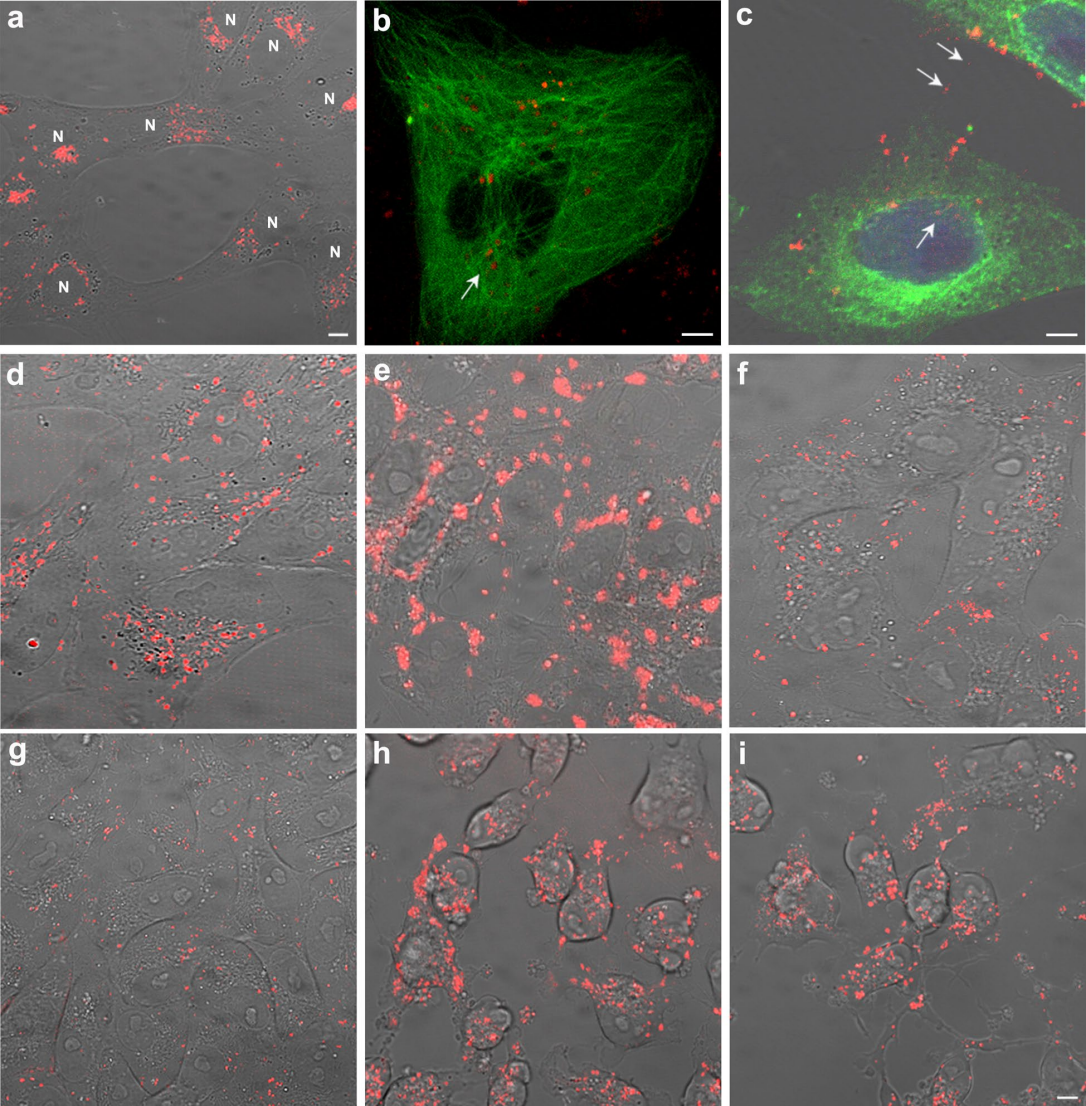
**a** Accumulation of Au@DynPro at the MTOC near the nucleus (N). **b** Progression of Au@DynPro towards the MTOC, or **c** nucleus and projections (arrows) in GFP-tubulin transfected cells. **d**–**i** Au@DynPro´s mobility was sensitive to microtubule-depolimerizing drugs. **d**, **g** Widespread cellular distribution of Au@DynPro before depolymerizing drug treatment, **e** Au@DynPro transport blockade and accumulation after 1 h drug incubation with 2.5 µM Nocodazole, and **f** mobility and dispersion recovery after washing. **g** Vero cells treated with 10 µM Au@DynPro and then **h** incubated for 1 h with 0.1 µM actin depolymerizing-drug LatrunculinA. This drug produced cell shrinkage because of actin cytoskeleton collapse that was not recovered after washing (**i**). However, Au@DynPro transport was still preserved within cells and projections. Scale bar: 10 µm

Linear movement of Au@DBPs, with occasional pauses and alternating directions, suggested that this was a microtubule-dependent transport. During the first minutes after incubation, Au@DBPs were rapidly internalized, moved and accumulated near the nucleus at the microtubule-organizing center (MTOC) (Fig. 4a). This preferential localization of Au@DBPs at the MTOC was found in non-polarized cells (Fig. 4b). Conversely, in polarized cells, movement was directed to the cell periphery and to cell projections (Fig. 4c). Linear movement of Au@DBPs was bidirectional, reached the cell periphery and projections being transported from one cell to another. These characteristics were found for all Au@DBPs tested.

Au@DBP dispersed distribution, movement and spread (Fig. 4d) was reversibly stopped with the microtubule depolymerizing agent Nocodazole (Fig. 4e). After washing and media replacement, movement and dispersion of Au@DBPs in the culture were recovered (Fig. 4f). In contrast, drugs that depolymerize the actin cytoskeleton, such as Latrunculin A, did not stop the motion of the Au@DBP between cells through projections, even under conditions of cell collapse after actin cytoskeleton depolimerization (Fig. 4g–i). This effect was not reversible by washing and media replacement as expected.

### Efficient transport of Au@DBP across the nuclear membrane

**Fig. 5:**
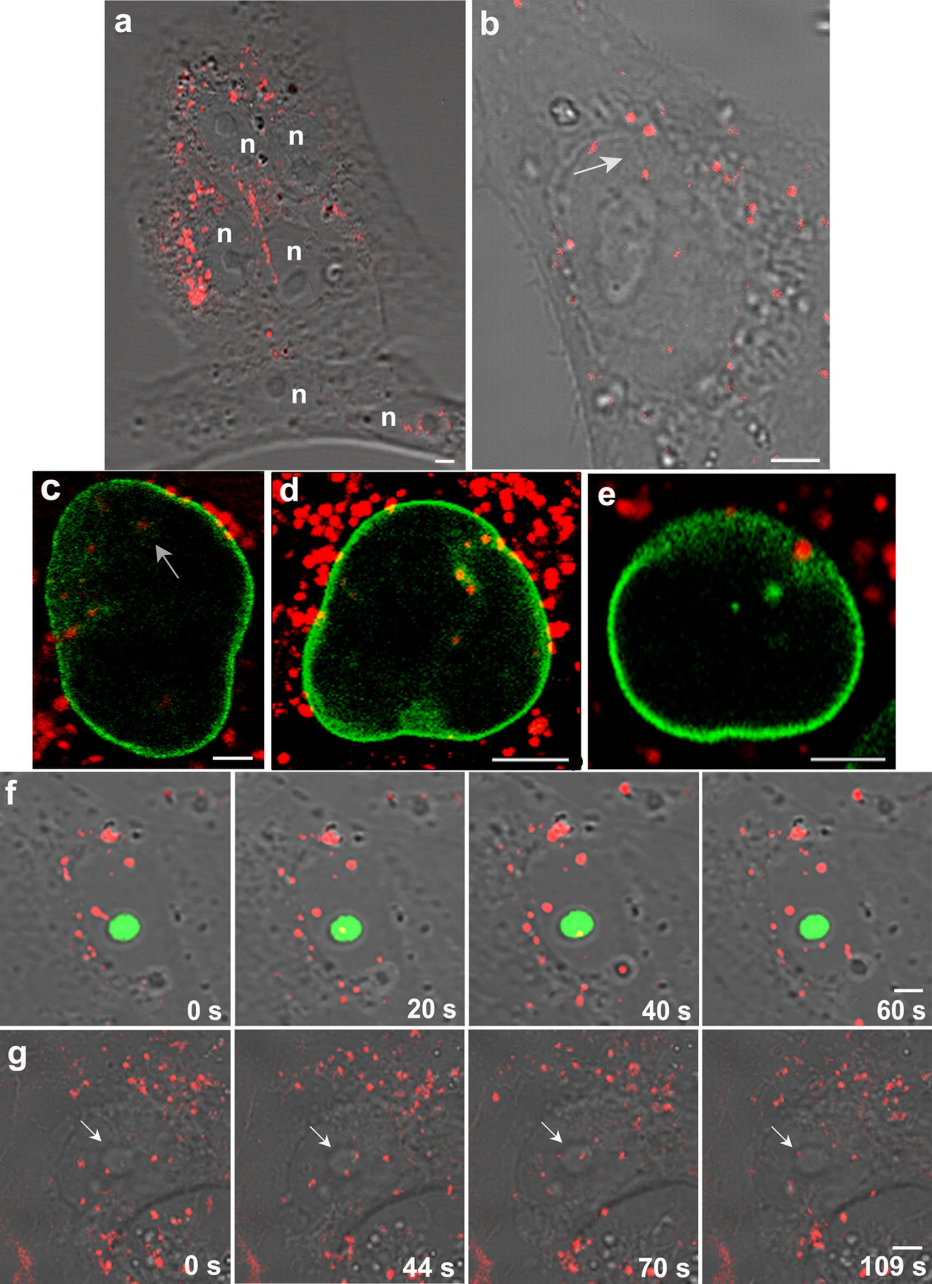
**a**, **b** Nuclear penetration capacity of Au@DynPro (n: nucleus). **c**–**e** Nuclear envelope appeared discontinuous as Au@DynPro shuttle the nucleus in Vero cells transfected with GFP-laminB receptor (**c**) and GFP-laminB1 (**d**, **e**) as shown in equatorial optical sections. Sample images show NPs entering the nucleus and nuclear lamina folding at sites of NPs entry. **f** Peptides shuttle the nucleus in Vero cells transfected with GFPB23 nucleolin (**g**) Au@DynPro in their way across the nucleus with visible imprints of their paths (**b**, arrow). Scale bar: 5 μm

Au@DBPs crossed the nuclear envelope and travelled the nuclear compartment (Fig. 5). This property was found for all the different Au@DBPs tested but not for the control Au@IntCt (Fig. 2c). Au@DBPs produced a visible imprint of their movement along the nucleus that was physically evident using bright field microscopy as linear protrusions as Au@DBPs moved through the nuclear area and the nucleolus (Fig. 5a, b, g).

Au@DBPs travelled across the nuclear envelope and the nuclear lamina, as shown in GFP-laminB receptor (Fig. 5c) and GFP-laminB1 transfected cells (Fig. 5d, e). In fact, Au@DBPs produced invaginations of the nuclear envelope at multiple sites (Figure 5c–e).

In the nucleolus, peptides colocalized with GFP-B23 nucleolin as they crossed the nucleolus, without affecting the morphology of this structure (Fig. 5f). Similarly, Au@DBPs entered nucleolus (Fig. 5g).

It is important to note that cell viability and cell proliferation were unaffected with any Au@DBP formulation (Additional file 1: Figure S5).

## Discussion

Molecular motors have previously allowed developing nanoscale transport systems powered by biomolecular motors or molecular shuttles [7]. Peptides binding to a cellular protein that is highly evolutionarily conserved (DYNLL1) [19] and a molecular hub building long-life molecular complexes in cells, made this transport very efficient in a number of cell lines tested, including mammalian cells and some of non-mammalian origin [21, 27, 40].

Here, the dynamic properties of DBPs were successfully translated into Au NPs (*i.e.*, Au@DBPs), which may be highly useful in the drug delivery area. These results are in agreement with previous reports that employed soft carriers, such as microspheres [41] or liposomes [42], functionalized with dynein light chain 8 (LC8) peptide binding motifs. Similarly, in the present work, we selected peptides able to bind a dynein chain with very high affinity [31], which were linked to hard Au@tiopronin NPs. In our study, non-significant differences regarding dynamic properties between Au@DBPs and “bare” DBPs were observed. Probably, this fact occurs due to the small size of our Au NPs, limiting the interaction of one dynein with one NP. Moreover, a previous theoretical work suggested that motor protein binding molecules attached to solid carriers work collectively rather than cooperatively [43].

NPs are small particles (from a few to 200 nm in diameter) especially useful for delivery as can be aerosolized [44], can be made highly biocompatible, allow site specific targeting to a specific cell population, permit controlled drug release and can be designed to biodegrade in an acceptable time span [35, 45]. The stability found for Au@DBPs and long times of persistence inside the cultured cells, make them suitable candidates for drug delivery. The Au@DBPs ability for spreading to neighboring cells remaining intracellular would improve efficacy and optimize dosage for compounds to be delivered.

Dynein has a central role in the formation of the mitotic spindle during mitosis and interacts dynamically with the nuclear envelope directing assembly and reassembly of nuclear lamins. The penetration capacity of Au@DBPs could rely on the ability of dynein to interact dynamically with the nuclear envelope and thus direct the disassembly of nuclear lamins as it occurs at early stages of mitosis [23, 25]. Other cell delivery mechanisms allow entering the nucleus efficiently only in dividing cells, because of nuclear envelope rearrangements naturally occurring at prometaphase. In contrast, Au@DBPs are virus-free delivery vehicles that elicited nuclear envelope reorganization also in non-dividing cells. Hence, Au@DBPs were able to take advantage of these properties, move in the crowded cellular environment and change the permeability of the nucleus compartment.

Targeting of drugs and NPs to tumors poses particular difficulties in delivery; drugs only penetrate few cells around blood vessels [46, 47]. This problem is particularly found in solid tumors, which are poorly perfused because blood vessels and lymphatics are usually dysfunctional. The leakiness of tumor vessels impairs enhanced permeability and retention effect of compounds delivered [48]. The capacity of Au@DBPs to be transferred from one cell to another could boost spreading of therapeutic NPs reaching neighboring cells. This would allow optimal dosage, increasing therapeutic effect and minimizing side effects.

## Conclusions

We have shown dynein-motor powered NP motility in the crowded cellular environment and across the nuclear envelope. The dynamic properties of these Au@DBPs made them especially appropriate to load compounds and achieve the desired requirements of intracellular delivery to all compartments. Moreover, the quick spreading of these functionalized NPs between cells, remaining intracellular, reduces exposure to the extracellular environment minimizing eventual loses. These characteristics, together with their intracellular accumulation capacity would in turn allow reductions on administration doses of a given drug or therapy because of its better biodisponibility in a handful of applications.

## Methods

### Peptides and cell lines

Peptide sequences spanned the DYNLL1 binding domain of viral proteins (Table 1), that is, African swine fever virus p54 (DynPro) and a shorter sequence including critical amino acids for binding (ShortPro), Rabies virus P protein (TransRb), and control non-dynein-binding amino acid sequence (IntCt). An octa-arginine tail was added to all these peptides, which were also labeled with TAMRA. Peptides were synthesized and purified by HPLC over 95% of purity by Genecust. The following cell lines were tested: Vero kidney epithelial cells (ATCC CCL-81), Madin-Darby Canine Kidney Epithelial Cells (MDCK; ATCC CCL-34), Human embryonic kidney cells transformed with adenovirus 5 (HEK293T/17; ATCC CRL-11268), and Epidermal cancer cells (HeLa; CCL-2). Cells were cultured in DMEM with FBS from Lonza. SK-N-MC human neuroblastoma cells were cultured in EMEM with pyruvate (ATCC HTB-10). These mammalian cells were incubated in sterile 35-mm culture dishes with 10 µM peptides, or 1.5 µM NPs, in a CO_2_ incubator at 37 °C for 1 h and then imaged.

### Chemicals for nanoparticle synthesis

All the chemicals were of reagent grade and were used without further purification. Hydrogen tetrachloroaureate (III) trihydrate (99.9%), (N-(3-dimethylaminopropyl)-N´-ethylcarbodiimide hydrochloride, Tris(hydroxymethyl) aminomethane (TRIS, > 98.8%) and 2-[N-morpholino] ethanesulfonic acid (99.5%) were purchased from Sigma-Aldrich; N-(2-mercaptopropionyl) glycine (> 98%) and N-hydroxysulfosuccinimide (> 97%) were purchased from Fluka and NaBH_4_ (98%) from Lancaster. Dynein binding peptides (DBPs, *i.e.* DynPro, ShortPro and TransRb) and the control peptide (IntCt) were purchased from Genecust; α-methoxy-ω-amino polyethylene glycol (CH_3_O-PEG-NH_2_, 750 Da) was obtained from Rapp-Polymere. Tetramethylrhodamine 5–carboxamide cadaverine (TAMRA-CAD) was purchase from Anaspec. Phosphate buffer saline (PBS) was obtained from Lonza. House distilled water was further purified using a Milli-Q reagent grade water system (Millipore). Buffers were prepared according to standard laboratory procedure. Other chemicals were reagent grade and used as received.

### Nanoparticle synthesis and characterization

#### Characterization

UV–Vis spectra were carried out with Varian Cary 50 spectrophotometer. Florescence spectra were carried out with a LS-55 Fluorescence Spectrometer. ζ-potential measurements were done using a ZetaPALS analyser from Brookhaven. All spectra were collected in MilliQ water; for ζ-potential characterization the aqueous solution was adjusted to 1 mM KCl. For TEM examinations, a single drop (5 µl) of the aqueous solution (0.1 mg ml^−1^) of the Au NPs was placed onto a copper grid coated with a carbon film. The grid was left to dry in air for several hours at room temperature. TEM analysis was carried out on a Tecnai 20 FEI microscope operated at 200 kV. Particle size distribution of the Au NPs was evaluated from several micrographs using a program for image processing and analysis (ImageJ).

#### Au@tiopronin

Hydrogen tetrachloroaureate(III) trihydrate (0.15 g, 0.4 mmol) and N-(2-mercaptopropionyl) glycine (tiopronin; 0.19 g, 1.2 mmol) were codissolved in 20 ml of 6:1 methanol/acetic acid, giving a ruby red solution. NaBH_4_ (0.30 g, 8.0 mmol) in 7.5 ml of H_2_O was added with rapid stirring. The black suspension that was formed was stirred for an additional 30 min after cooling, and the solvent was then removed under vacuum at 40 °C. The crude Au@tiopronin was completely insoluble in methanol but quite soluble in water. It was purified by dialysis, in which the pH of 130 mg of crude product dissolved in 20 ml of water was adjusted to 1 by dropwise addition of concentrated HCl. This solution was loaded into 15 cm segments of seamless cellulose ester dialysis membrane (Sigma, MWCO 10 kDa), placed in 4 l beakers of water, and stirred slowly, recharging with fresh water approximately every 10 h over the course of 72 h. The dark blue Au@tiopronin solutions were collected from the dialysis tubes and were lyophilized. Yield: 96 mg.

#### Au@peptides/TAMRA-CAD/PEG

N-(3-Dimethylaminopropyl) -N´-ethylcarbodiimide hydro-chloride (EDC; 1 mg, 5 µmol) and N-hydroxysulfosuccinimide (sulfo NHS; 3 mg, 13.5 µmol) were added to 1 ml of Au@tiopronin (1 mg) in 2-[N-morpholino] ethanesulfonic acid (MES) (50 mM, pH 6.5). NPs were left to react with EDC (carbodiimide activation) over 30 min at 37 °C under mild stirring conditions. NPs were then purified of excess EDC and sulfo NHS) over a PD-10 column (GE Healthcare). The peptide DynPro (27.5 nmol) or ShortPro (27.5 nmol) or TransRb (27.5 nmol) or IntCt3 (27.5 nmol) or TAMRA-CAD (55 nmol) were added and the mixture was stirred over 1 h at room temperature before the addition of CH_3_O-PEG-NH_2_ (750 Da; 2.6 µmol) for coupling to remaining activated carboxylic groups; then samples were left under mild stirring conditions at 4 °C overnight. Then, this solution was loaded into centrifugal filters (Amicon Ultra-0.5 ml, MWCO 10 kDa) for three consecutive steps of purification of potentially uncoupled peptide, TAMRA or PEG, and concentration of the mixture prior to suspension in water. For preventing the possible absorption (non-covalently attached to the NPs) of TAMRA or any of the peptides, samples (0.5 ml at 2 mg mL^−1^) were loaded into ca. 1 cm segments of seamless cellulose ester dialysis membrane (Sigma, MWCO 10 kDa), placed in 1 l beakers of a solution 1 M NaCl in MilliQ water, and stirred slowly over the course of 72 h, recharging with fresh saline solution approximately every 10 h. Then, samples were desalted over a PD-10 column, concentrated with centrifugal filters (Amicon Ultra-0.5 ml, MWCO 10 kDa), suspended in PBS (for cell cultures) or water (for characterization) at 2 mg ml^−1^, and filtrated over 0.22 µm cellulose filters for sterilization. ζ-potential measurements are shown in Additional file 1: Table S1; UV–Vis and fluorescence spectra are shown in Additional file 1: Figures S1 and S2.

Control samples were prepared for all of the peptide types and TAMRA using the same conditions with the exception of EDC and S-NHS, aiming to prove that unspecific absorption of the peptides on the NPs was avoided. Theses samples were cleaned as the activated samples and they were characterized by UV/Vis and fluorescence spectroscopy, showing the absence of observable unspecific absorption (*i.e.*, lack of fluorescence).

### Incubation of cells with nanoparticles

All the experiments were carried out under sterile conditions working with microbiological class II safety cabinets. The NPs were resuspended in H_2_O, with a degree of purity milliQ and sterile, to obtain stock solutions at different concentrations. Special attention was paid to avoid turbidity in the solution and tips were always used with a filter to avoid possible cross contaminations. 500 µl aliquots were stored at 4 °C until the time of use. Working solutions of the NPs were made from stock solutions in DMEM SC medium (10% Foetal bovine serum (FBS), 1% penicillin/streptomicyn (PS), 1% Glutamine (G)) and adjusted to 0.2 mg/ml (1.5 μM NPs). The maximum number of NPs used per cell was estimated to be 2.7 × 10^9^ NPs/cell, that is, 300 μl of a solution of 1.5 μM NPs that was added to 10^5^ cells. In order to perform in vitro assays, Vero cells were cultured in 35 mm glass plates overnight. The cells were washed in DMEM SC (10% FBS, 1% PS, 1% G) and the existing medium was replaced by 300 ml of the solutions containing the NPs at optimal concentration of 1.5 μM. The NPs were incubated in incubation chambers at 37 °C and 5% CO2 and observed with the confocal microscope. The incubation time periods ranged from 20 min to 3 h to observe different degree of accumulation in the cells.

### Plasmids and transfections

A vector encoding enhanced green fluorescent protein (EGFP)-LBR (Lamin B receptor) was kindly provided by Loren Fong from UCLA. Plasmid encoding EGFP-Lamin B1 was a generous gift of Howard Worman from Columbia University New York. EGFP-tubulin and –actin, were from Clontech. A vector encoding B23 nucleolar protein was kindly provided by Carmen Rivas from Centro Nacional de Biotecnología, Madrid.

Transfections were performed by using the TransIT 2020 Transfection Reagent from Mirus according to the manufacturer’s recommendations. Briefly, Vero cells were grown on 35 mm tissue culture plates, in DMEM (5% FBS, 1% PS, 1% G), until 80% confluence. Separately, 50 µl of DMEM, serum- and antibiotics-free, was mixed with 0.5 µg of DNA and 1.3 µl of TransIT 2020. The mixture was incubated for 20 min at room temperature before addition to cells. To minimize cytotoxicity and increase the efficiency of the transfection, cells medium was replaced by 300 µl of fresh DMEM 5% serum and antibiotics-free before adding the DNA-TransIT mixture. Similarly, after 4 h, the transfection mixture was removed from cells and 500 µl of fresh medium (5% FBS, 1% PS, 1% G) was added. At 24 h after transfection, cells were incubated with the NPs as explained below and analyzed by confocal microscopy.

### Inhibition assays

Nocodazole was used at concentration of 2.5 µM and actin depolymerizing Latrunculin A at 0.1 µM. We first ensured that cytotoxicity, determined by the trypan blue exclusion method, did not exceed 10% of cell death after drug incubation at the indicated working concentrations. Cells were pretreated for 30 min with inhibitors at the indicated working concentrations in growth medium for 30 min at 37 °C, further incubated with 1.5 µM of NPs for 30 min at 37 °C and then imaged. After 2 h, culture medium was replaced and washed for 1 h and then imaged.

### Cell viability and proliferation assays

To evaluate cell viability after incubation with peptides and NPs, Vero cells seeded in 24 well plates were incubated in DMEM containing delivery DBP or negative control IntCt at concentrations ranging from 0 to 100 µM of each peptide. For NPs the concentrations were ranging from 0 to 1 mg/ml (1–7.5 µM NPs). After incubation with peptides or nanoparticles for 24 h, cells were harvested and the number of viable cells present in the cell suspensions was determined by Tripan blue dye exclusion assay (Sigma). Briefly, 20 µl of PBS with Tripan Blue 0.08% (w/v) were added to equal volume of cell suspension and mixed. After 2 min, blue cells (dead cells) were counted using a hematocytometer and a conventional light microscope.

To evaluate cell proliferation 3 × 10^4^ Vero cells/well seeded in 96 well plates were incubated in 50 µl DMEM containing DBPs and negative control or NPs at the range of concentrations previously used in the cell viability assays. After 24 h incubation, cell proliferation was determined using CellTiter 96 Aqueous™ (Promega) assay, following manufacturer´s indications.

### Flow cytometry

Vero cells seeded in 24-well plates were incubated for 120 min at 37 °C with Au@DBPs at several concentrations. Then, cells were washed with PBS, harvested by trypsinization and washed again with flow cytometry buffer (PBS, 0.01% sodium azide and 0.1% bovine serum albumin). In order to determine the fluorescence intensity per dose, 10,000 cells/per each Au@DBPs concentration were scored and analysed in a FACS Canto II flow cytometer (BD Sciences).

### Time-lapse video microscopy

Confocal microscopy was carried out using a Leica TCS SPE confocal microscope that included a humidified incubation chamber, a CO_2_ controller and a heating unit. Selected stacks were recorded every 10-s using the Leica Microsystems LAS AF program and the films were displayed at 1–5 frames/s.

### Fluorescence quantification

To quantify the intracellular accumulation of different DBP, cytoplasmic and background regions were selected and mean fluorescence intensity (MFI) quantified. Final MFI was calculated as indicated (cytoplasmic region—background). Quantitative analysis of MFI was performed with the Leica LAS-AF imaging program. At least thirty cells from three independent experiments were examined for each formulation.

### Data analysis

One-way analysis of variance was performed with the statistical package GraphPad InStat. Bonferroni’s correction was applied for multiple comparisons. Data were presented as mean standard deviations. Differences were considered statistically significant with a *p* value of 0.001 (α = 0.05).

## Additional files

**Additional file 1: Figure S1.** UV/Vis spectra of “bare” NPs (Au@tiopronin) before the modification, and peptide, PEG or TAMRA-CAD modified NPs. a) DBP (red line, Au@tiopronin-DynPro), IntCT (cyan line, Au@tiopronin-IntCt) and TAMRA (blue line, Au@tiopronin-IntCt-TAMRA-CAD); b) DBP-PEG (red line, Au@tiopronin-DynPro/PEG), IntCT-PEG (cyan line, Au@tiopronin-IntCt/PEG) and TAMRA-PEG (blue line, Au@tiopronin-IntCt-TAMRA-CAD/PEG). **Figure S2.** Fluorescence spectra of “bare” NPs (Au@tiopronin) before the modification, and peptide, PEG or TAMRA-CAD modified NPs. a) DBP (red line, Au@tiopronin-DynPro), IntCT (cyan line, Au@tiopronin-IntCt) and TAMRA (blue line, Au@tiopronin-IntCt-TAMRA-CAD); b) DBP-PEG (red line, Au@tiopronin-DynPro/PEG), IntCT-PEG (cyan line, Au@tiopronin-IntCt/PEG) and TAMRA-PEG (blue line, Au@tiopronin-IntCt-TAMRA-CAD/PEG).** Figure S3.** ζ-potential bar diagram of “bare” NPs (Au@tiopronin) before the modification, and modified peptide or TAMRA-CAD modified NPs: DBP (Au@DynPro), IntCT (Au@IntCt), TAMRA (Au@TAMRA-CAD), DBP-PEG (Au@DynPro/PEG), IntCT-PEG (Au@IntCt/PEG) and TAMRA-PEG (Au@TAMRA-CAD/PEG).** Table S1.** ζ-potential values of “bare” NPs (Au@tiopronin) before the modification, and peptide, PEG or TAMRA-CAD modified NPs: DBP (Au@DynPro), IntCT (Au@IntCt), TAMRA (Au@TAMRA-CAD), DBP-PEG (Au@DynPro/PEG), IntCT-PEG (Au@IntCt/PEG) and TAMRA-PEG (Au@TAMRA-CAD/PEG).** Figure S4.** The figure shows the cellular uptake (according to intracellular MFI) of Au@DynPro, Au@DynPro-PEG, compared to nanoparticles modified with internal control peptide (IntCt), Au@IntCt and Au@IntCt-PEG. Mean fluorescence intensity (MFI) of modified NPs after 1 h incubation.** Figure S5.** This figure shows the absence of cytotoxic effect of the NPs in Vero cells. Cell viability (a) and cell proliferation (b) were analyzed after incubation of cells with increasing concentrations of the different Au@DBP that exceeded those used in this study. A significant decrease in cell counts or cell proliferation was not observed.

**Additional file 2: Movie 1.** Motion beyond cell boundaries. This movie shows a general view of Au@DBP motion and dispersion in 293T cells incubated with 0.2 mg/ml of Au@DynPro during 1.5 h. Au@DBP displayed short and long tracks of bidirectional motion along a cell projection connecting a neighboring cell. Movement results in transfer of Au@DynPro to the latter. The time lapse covers about 7 min at a rate of 5 frames/s.

**Additional file 3: Movie 2.** Intracellular movement of NPs linear trajectories. This movie displays a Vero cell incubated with Au@DynPro at 0.2 mg/ml during 1.5 h showing linear and stable tracks of directed motion in the perinuclear area. The time lapse covers 40 s at a rate of 3 frames/s.
