# Supplementary material for: Nanoparticles engineered to bind cellular motors for efficient delivery
Source: J Nanobiotechnology. 2018 Mar 30;16:33. doi: 10.1186/s12951-018-0354-1 (PMC5877387; doi:10.1186/s12951-018-0354-1)
Supplement: Supplementary file 1 — Additional file 1: Figure S1. UV/Vis spectra of “bare” NPs (Au@tiopronin) before the modification, and peptide, PEG or TAMRA-CAD modified NPs. a) DBP (red line, Au@tiopronin-DynPro), IntCT (cyan line, Au@tiopronin-IntCt) and TAMRA (blue line, Au@tiopronin-IntCt-TAMRA-CAD); b) DBP-PEG (red line, Au@tiopronin-DynPro/PEG), IntCT-PEG (cyan line, Au@tiopronin-IntCt/PEG) and TAMRA-PEG (blue line, Au@tiopronin-IntCt-TAMRA-CAD/PEG). Figure S2. Fluorescence spectra of “bare” NPs (Au@tiopronin) before the modification, and peptide, PEG or TAMRA-CAD modified NPs. a) DBP (red line, Au@tiopronin-DynPro), IntCT (cyan line, Au@tiopronin-IntCt) and TAMRA (blue line, Au@tiopronin-IntCt-TAMRA-CAD); b) DBP-PEG (red line, Au@tiopronin-DynPro/PEG), IntCT-PEG (cyan line, Au@tiopronin-IntCt/PEG) and TAMRA-PEG (blue line, Au@tiopronin-IntCt-TAMRA-CAD/PEG). Figure S3. ζ-potential bar diagram of “bare” NPs (Au@tiopronin) before the modification, and modified peptide or TAMRA-CAD modified NPs: DBP (Au@DynPro), IntCT (Au@IntCt), TAMRA (Au@TAMRA-CAD), DBP-PEG (Au@DynPro/PEG), IntCT-PEG (Au@IntCt/PEG) and TAMRA-PEG (Au@TAMRA-CAD/PEG). Table S1. ζ-potential values of “bare” NPs (Au@tiopronin) before the modification, and peptide, PEG or TAMRA-CAD modified NPs: DBP (Au@DynPro), IntCT (Au@IntCt), TAMRA (Au@TAMRA-CAD), DBP-PEG (Au@DynPro/PEG), IntCT-PEG (Au@IntCt/PEG) and TAMRA-PEG (Au@TAMRA-CAD/PEG). Figure S4. The figure shows the cellular uptake (according to intracellular MFI) of Au@DynPro, Au@DynPro-PEG, compared to nanoparticles modified with internal control peptide (IntCt), Au@IntCt and Au@IntCt-PEG. Mean fluorescence intensity (MFI) of modified NPs after 1 h incubation. Figure S5. This figure shows the absence of cytotoxic effect of the NPs in Vero cells. Cell viability (a) and cell proliferation (b) were analyzed after incubation of cells with increasing concentrations of the different Au@DBP that exceeded those used in this study. A significant decrease in cell counts or cell proliferatio [file 12951_2018_354_MOESM1_ESM.docx]

**NANOPARTICLES ENGINEERED TO BIND CELLULAR MOTORS FOR EFFICIENT DELIVERY**

Inmaculada Dalmau, Pablo del Pino, Beatriz Pelaz, Miguel Ángel Cuesta-Geijo, Inmaculada Galindo, Maria Moros, Jesús M. de la Fuente and Covadonga Alonso

**ADDITIONAL INFORMATION**

**TABLE OF CONTENTS:**

**S1: UV/Vis spectra of nanoparticles**

**S2: Fluorescence spectra of nanoparticles**

**S3: ζ–potential of nanoparticles**

**S4: Effect of PEGylation of NPs in cell uptake**

**S5: Cell viability and proliferation of Vero cells incubated with nanoparticles**

**S6: Supporting Movie Legends**

**S1: UV/Vis spectra of nanoparticles**

After their synthesis, modification and purification, the modified Au@tiopronin NPs were characterized by UV-visible absorption spectroscopy (Varian Cary 5 UV-Vis-NIR spectrophotometer), *cf.* Figure S1.

**Figure S1.** UV/Vis spectra of “bare” NPs (Au@tiopronin) before the modification, and peptide, PEG or TAMRA-CAD modified NPs. a) DBP (red line, Au@tiopronin-DynPro), IntCT (cyan line, Au@tiopronin-IntCt) and TAMRA (blue line, Au@tiopronin-IntCt-TAMRA-CAD); b) DBP-PEG (red line, Au@tiopronin-DynPro/PEG), IntCT-PEG (cyan line, Au@tiopronin-IntCt/PEG) and TAMRA-PEG (blue line, Au@tiopronin-IntCt-TAMRA-CAD/PEG)

**S2: Fluorescence spectra of nanoparticles**

Fluorescence spectra results using as λ _excitation_= 550 nm are shown in Figure S2.

**Figure S2.** Fluorescence spectra of “bare” NPs (Au@tiopronin) before the modification, and peptide, PEG or TAMRA-CAD modified NPs. a) DBP (red line, Au@tiopronin-DynPro), IntCT (cyan line, Au@tiopronin-IntCt) and TAMRA (blue line, Au@tiopronin-IntCt-TAMRA-CAD); b) DBP-PEG (red line, Au@tiopronin-DynPro/PEG), IntCT-PEG (cyan line, Au@tiopronin-IntCt/PEG) and TAMRA-PEG (blue line, Au@tiopronin-IntCt-TAMRA-CAD/PEG).

**S3: ζ–potential of nanoparticles**

ζ-potential measurements were performed at pH 6.9, in 1 mM KCl solution after sample filtration with 0.2 µm cellulose filters. Results are summarized in Figure S3 in a bar graph and in Table S1.

**Figure S3.** ζ–potential bar diagram of “bare” NPs (Au@tiopronin) before the modification, and modified peptide or TAMRA-CAD modified NPs: DBP (Au@DynPro), IntCT (Au@IntCt), TAMRA (Au@TAMRA-CAD), DBP-PEG (Au@DynPro/PEG), IntCT-PEG (Au@IntCt/PEG) and TAMRA-PEG (Au@TAMRA-CAD/PEG).

**Table S1.** ζ–potential values of “bare” NPs (Au@tiopronin) before the modification, and peptide, PEG or TAMRA-CAD modified NPs: DBP (Au@DynPro), IntCT (Au@IntCt), TAMRA (Au@TAMRA-CAD), DBP-PEG (Au@DynPro/PEG), IntCT-PEG (Au@IntCt/PEG) and TAMRA-PEG (Au@TAMRA-CAD/PEG).

| **Sample** | **ζ-potential (mV)** |
| --- | --- |
| **“bare”** | -40.0 ± 5.0 |
| **TAMRA** | -3.5 ± 2.0 |
| **TAMRA-PEG** | -0.1 ± 0.1 |
| **DBP** | -15.0 ± 8.0 |
| **DBP-PEG** | -1.5 ± 1.0 |
| **IntCt** | -36.3 ± 4.0 |
| **IntCt-PEG** | -30.0 ± 5.5 |

**S4: Effect of PEGylation of NPs in cell uptake**

**
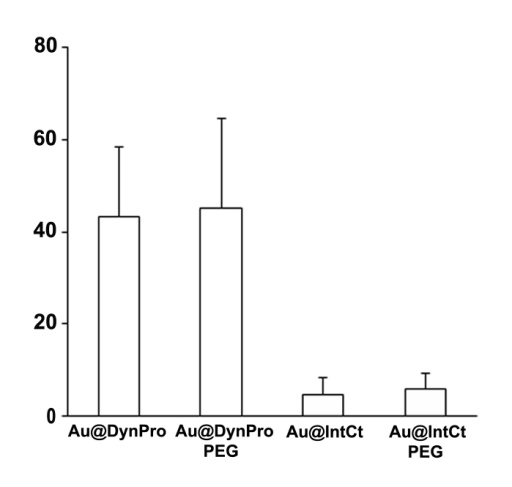
**

**Figure S4.** The figure shows the cellular uptake (according to intracellular MFI) of Au@DynPro, Au@DynPro-PEG, compared to nanoparticles modified with internal control peptide (IntCt), Au@IntCt and Au@IntCt-PEG. Mean fluorescence intensity (MFI) of modified NPs after 1 h incubation.

**S5: Cell viability and proliferation of Vero cells incubated with nanoparticles**


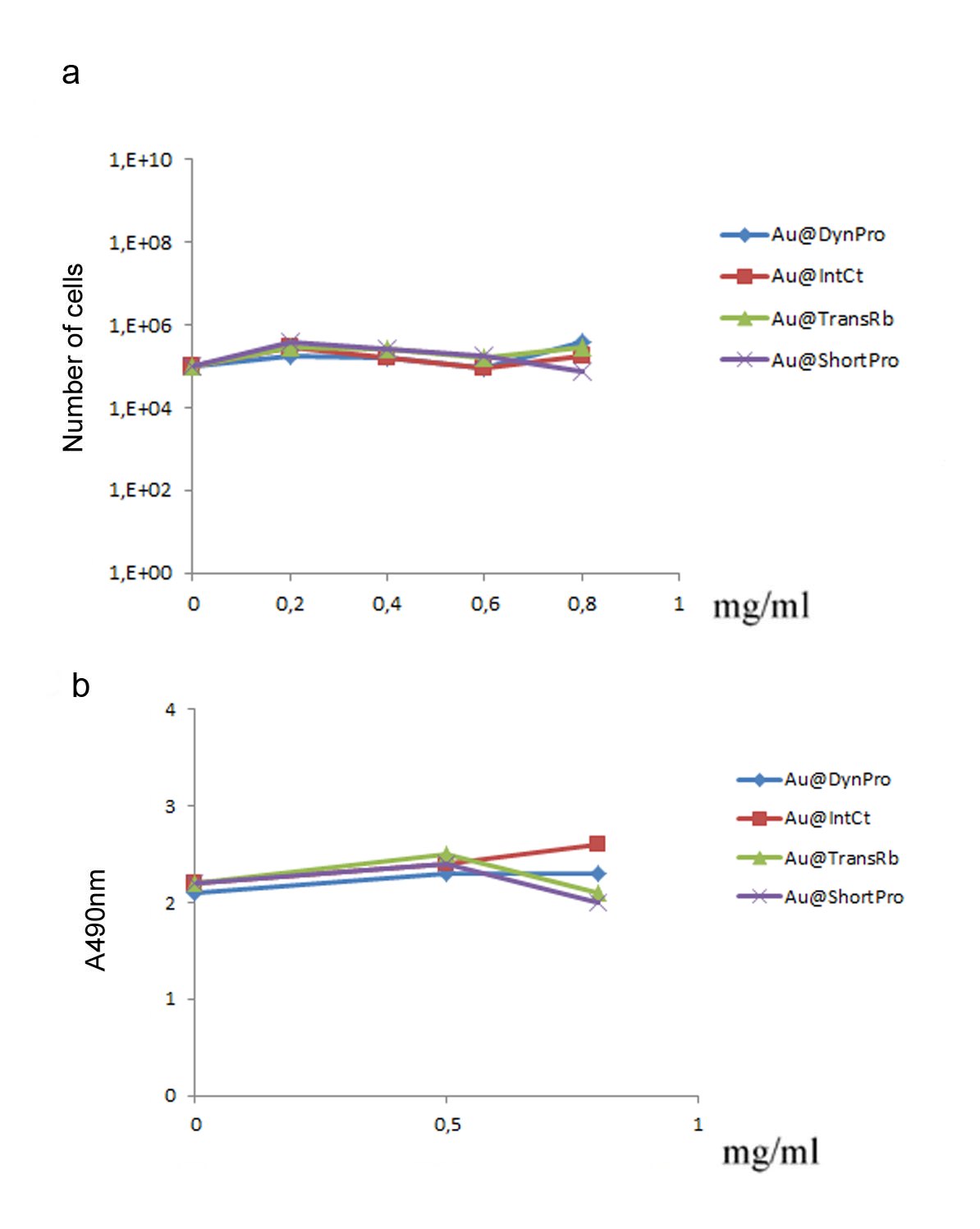


**Figure S5.** This figure shows the absence of cytotoxic effect of the NPs in Vero cells. Cell viability (a) and cell proliferation (b) were analyzed after incubation of cells with increasing concentrations of the different Au@DBP that exceeded those used in this study. A significant decrease in cell counts or cell proliferation was not observed in any case.

**S6**. **Supporting Movie Legends**

**Movie 1: Motion beyond cell boundaries.** This movie shows a general view of Au@DBP motion and dispersion in 293T cells incubated with 0.2 mg/ml of Au@DynPro during 1.5 h. Au@DBP displayed short and long tracks of bidirectional motion along a cell projection connecting a neighboring cell. Movement results in transfer of Au@DynPro to the latter. The time lapse covers about 7 min at a rate of 5 frames/sec.

**Movie 2: Intracellular movement of NPs linear trajectories.** This movie displays a Vero cell incubated with Au@DynPro at 0.2 mg/ml during 1.5 h showing linear and stable tracks of directed motion in the perinuclear area. The time lapse covers 40 seconds at a rate of 3 frames/sec.
